# Mutagenesis of the Aquaporin 4 Extracellular Domains Defines Restricted Binding Patterns of Pathogenic Neuromyelitis Optica IgG*

**DOI:** 10.1074/jbc.M115.647149

**Published:** 2015-03-19

**Authors:** Gregory P. Owens, Alanna Ritchie, Andrea Rossi, Kristin Schaller, Scott Wemlinger, Hannah Schumann, Andrew Shearer, Alan S. Verkman, Jeffrey L. Bennett

**Affiliations:** From the Departments of ‡ Neurology and; ‖ Ophthalmology and; ** Neuroscience Program, University of Colorado at Denver, Anschutz Medical Campus, Aurora, Colorado 80045,; the § Department III-Developmental Genetics, Max Planck Institute for Heart and Lung Research, Ludwigstrasse 43, 61231 Bad Nauheim, Germany, and; the ¶ Departments of Medicine and Physiology, University of California, San Francisco, San Francisco, California 94143

**Keywords:** Aquaporin, Autoimmunity, Epitope Mapping, Monoclonal Antibody, Neuroimmunology, Demyelination, Neuromyelitis Optica

## Abstract

Neuromyelitis optica-immunoglobulin G (NMO-IgG) binds to aquaporin-4 (AQP4) water channels in the central nervous system leading to immune-mediated injury. We have previously demonstrated that a high proportion of CSF plasma cells of NMO patients produce antibody to the extracellular domains of the AQP4 protein and that recombinant IgG (rAb) derived from these cells recapitulate pathogenic features of disease. We performed a comprehensive mutational analysis of the three extracellular loops of the M23 isoform of human AQP4 using both serial and single point mutations, and we evaluated the effects on binding of NMO AQP4-reactive rAbs by quantitative immunofluorescence. Whereas all NMO rAbs required conserved loop C (^137^TP^138^ and Val^150^) and loop E (^230^HW^231^) amino acids for binding, two broad patterns of NMO-IgG recognition could be distinguished based on differential sensitivity to loop A amino acid changes. Pattern 1 NMO rAbs were insensitive to loop A mutations and could be further discriminated by differential sensitivity to amino acid changes in loop C (^148^TM^149^ and His^151^) and loop E (Asn^226^ and Glu^228^). Alternatively, pattern 2 NMO rAbs showed significantly reduced binding following amino acid changes in loop A (^63^EKP^65^ and Asp^69^) and loop C (Val^141^, His^151^, and Leu^154^). Amino acid substitutions at ^137^TP^138^ altered loop C conformation and abolished the binding of all NMO rAbs and NMO-IgG, indicating the global importance of loop C conformation to the recognition of AQP4 by pathogenic NMO Abs. The generation of human NMO rAbs has allowed the first high resolution mapping of extracellular loop amino acids critical for NMO-IgG binding and identified regions of AQP4 extracellular structure that may represent prime targets for drug therapy.

## Teaser

**Background:** Patient-derived aquaporin 4 (AQP4)-specific recombinant monoclonal antibodies (rAbs) cause neuromyelitis optica (NMO)-specific nervous system injury in animal models.

**Results:** AQP4-specific rAbs bind human AQP4 based on differential sensitivity to loop A and C mutations.

**Conclusion:** AQP4-specific rAbs derived from NMO patients recognize multiple conformational epitopes within the extracellular domains of human AQP4.

**Significance:** High resolution mapping of AQP4 autoantibody epitopes identifies target regions for potential blocking therapies.

## Introduction

Neuromyelitis optica (NMO)^2^ is a severe inflammatory demyelinating disorder of the central nervous system (CNS) that preferentially targets optic nerves and spinal cord leading to paralysis and vision loss. Although once considered a variant of multiple sclerosis, clues to the nature of disease were first revealed by the detection of an NMO-specific serum antibody (Ab) response, termed NMO-IgG, that immunostained the surface of microvessels, pia, subpia, and Virchow-Robin spaces in the CNS (1). NMO IgG was subsequently shown to recognize the water channel aquaporin-4 (AQP4), which is expressed on astrocytes in the CNS and is preferentially polarized to astrocytic end-foot processes (2). We showed that during disease exacerbation, CSF from NMO patients contained a dynamic population of expanded and clonally related plasmablasts that were enriched for cells producing Abs against the extracellular domains of AQP4 (3). More importantly, AQP4-specific recombinant Abs (rAbs) derived from these CSF plasmablasts, when administered in experimental animals, recapitulated the myriad pathological features of NMO lesions that include perivascular loss of astrocytes, terminal complement activation, granulocyte infiltration, and subsequent oligodendrocyte cell death and myelinolysis (3–5). The pathology produced in animal models parallels that observed with serum-derived NMO-IgG, indicating that CSF rAbs reproduce the disease-specific response of AQP4-seropositive patients (5–7).

Although knowledge of NMO pathology has been greatly enhanced by the identification of NMO-IgG, many questions remain regarding the origin of this CNS B cell response, its variation across the NMO patient population, and the steps and signaling pathways leading from astrocyte destruction to demyelination. One critical factor that promotes Ab-mediated CNS tissue damage is the ability of the M23 isoform of AQP4 to assemble from tetramers into large supramolecular structures called orthogonal array of particles (OAPs). AQP4 is expressed in alternative isoforms termed long (M1) and short (M23). The M23 isoform promotes OAP formation, and the size of OAPs is determined by the ratio of M23:M1 isoforms (8, 9). A quantitative comparison of binding to the M1 and M23 isoforms shows that both serum NMO-IgG and most NMO CSF-derived rAbs bind with higher affinity to OAPs than to tetramers and that structural changes in the AQP4 epitope upon array assembly, and not bivalent binding of IgG, drive this increased affinity (10). OAP formation is also required for anti-AQP4 Abs to initiate complement-dependent cell lysis, which likely occurs through the promotion of a multivalent interaction of C1q molecules with cell surface-bound AQP4 Abs (10, 11).

Presently, it is not known whether the binding of Abs to specific epitopes on AQP4 may preferentially affect lesion formation and contribute to differences in disease severity. Studies to define the depth and specificity of the NMO serum IgG response have been somewhat disparate. In two separate studies using serum NMO-IgG to define AQP4 epitopes, Pisani *et al.* (12) identified two major conformational AQP4 epitopes in the extracellular domains of AQP4 by mutagenesis of amino acids within the extracellular domains, whereas Iorio *et al.* (13) reported a broader array of AQP4 epitopes displayed on denatured AQP4 monomers, tetramers, and OAPs. Recently, mutation of Asp^69^ has been shown to alter loop A conformation and reduce binding of NMO patient sera (14). Each of these studies, however, was hampered by the use of polyclonal serum NMO-IgG that contains multiple AQP4 autoantibodies with a wide range of affinities directed against many distinct epitopes, some of which may be nonpathogenic species directed against denatured AQP4 protein or protein fragments. In this study, we define the dominant epitopes recognized by a panel of AQP4-specific monoclonal recombinant Abs from CSF plasmablasts recovered from five NMO patients. Site-directed mutagenesis was used to generate seven 4- to 6-amino acid serial substitutions and 29-point mutations encompassing most amino acids (glycines excluded) within each of the three extracellular loops (A, C, and E) of the M23 isoform of human AQP4. The effects of each mutation on OAP array formation and AQP4 rAb binding were evaluated to define distinct and not previously appreciated patterns of antigen recognition.

## EXPERIMENTAL PROCEDURES

### 

#### 

##### Patients

CSF was obtained from five NMO-IgG seropositive patients 1–8 weeks after the onset of unilateral monosymptomatic optic neuritis or transverse myelitis as part of their standard clinical care. The clinical testing of each patient's serum for NMO-IgG was performed at the Mayo Clinic Laboratories, Rochester, MN, using their standardized commercial clinical assay. Informed consent was obtained for all individuals prior to participation in this study.

##### CSF Cell Labeling and FACS

CSF cell collection, fluorescent labeling, and cell sorting of CD138^+^ CSF plasma cells and plasmablasts were performed as described (3). Cells were first selected in the size range of lymphocytes and plasma cells by forward and side light scattering. CD138^+^ cells were then identified and sorted into single wells of a 96-well PCR plate containing 20 μl of 1× RT buffer.

##### cDNA Synthesis and Amplification of VH and VL Chain Sequences

cDNA synthesis, nested PCR amplification, and purification of PCR products were performed as described (3, 15). Purified PCR products were sequenced at the University of Colorado Cancer Center DNA Sequencing Core. Sequences were analyzed and edited with 4Peaks software (Mek&Tosj.com) and then aligned to functional human immunoglobulin germline sequences using IMGT/V-QUEST.

##### Construction, Expression, and Purification of rAbs

rAbs were produced from NMO CSF plasma cell and plasmablast clonal populations using a dual vector transient transfection system. VH and VL PCR products were cloned into the expression vectors pIgG1FLAG and pCEP4, respectively, as described. Final constructs were sequenced and verified. Constructs were co-transfected into HEK293 cells using Lipofectamine 2000 (Invitrogen). After transfection, the cells were grown for 6–7 days in DMEM + 10% fetal bovine serum (FBS); the supernatant was harvested, fresh medium was added, and cells were propagated for 6–7 days. The cell culture supernatant was subsequently removed and combined with the previous collection. The cell culture supernatant was centrifuged at 10,000 × *g* to pellet cells and debris. Cell-free supernatants were passed twice through protein A-Sepharose (Sigma), and rAbs were eluted in 0.1 m glycine, 1 m NaCl, pH 3.0, and neutralized by the addition of 0.1 m Tris-HCl, pH 8.0. Recombinant IgG was subsequently exchanged and concentrated in storage buffer (PBS + 0.1% IgG/protease-free BSA) using Ultracell YM-30 microconcentrators (EMD Millipore, Darmstadt, Germany). Antibody integrity was confirmed by nondenaturing gel electrophoresis, and the IgG concentration was determined by a human IgG capture ELISA or by BCA colorimetric protein quantification (Pierce). The specificity of each rAb used in this study was confirmed either by FACS or by fluorescence immunostaining of mock- and AQP4-expressing cells (3).

##### Mutagenesis of M23-AQP4

Both 4–6 amino acid serial alanine or glycine substitutions and point mutations were introduced into M23 AQP4 extracellular loops using the PCR-based GeneArt site-directed mutagenesis system (Invitrogen) (Table 1). Partially overlapping sense and antisense primers were designed from the M23 AQP4 sequence with the desired nucleotide mutation(s) located near the center of each primer sequence. Methylation and mutagenesis reactions of AQP4 DNA were done using 20–25 ng of target AQP4 plasmid DNA according to the manufacturer's instructions. AQP4 DNA mutations were confirmed by sequencing, and endonuclease-free plasmid DNA was purified for transfection into mammalian cells (Qiagen catalog no. 12362).

Plasmid DNA containing M23-AQP4 or mutated M23-AQP4 was then transfected with Lipofectamine 2000 into U-87MG or HEK293 EBNA cells growing on poly-l-ornithine-treated coverslips. Cells were incubated for ∼24 h at 37 °C, rinsed in PBS, and fixed in 4% paraformaldehyde in PBS. Coverslips were stored at −20 °C until used.

##### Generation of Stable U-87MG Cell Lines Expressing AQP4M23 and Mutated AQP4M23

Plasmid DNA containing the AQP4-M23 cDNA sequence or the loop A mutant, ΔA3, cDNA sequence were linearized with PvuI for 1 h at 37 °C and then transfected into U-87MG astrocytoma cells with Lipofectamine 2000 per manufacturer's protocol. At 24 h post-transfection, G418 was added at a concentration of 400 μg/ml, and cells were monitored for survival. After ∼1 week, growing clonal populations were diluted in U-87MG selection media (DMEM high glucose media containing 10% FCS, 100 μg/ml penicillin/streptomycin, and 300 μg/ml G418) to less than 10 cells/ml, and 100 μl were deposited into a 96-well plates. Plates were monitored 1–2 weeks for clonal growth and then 5–6 individual wells were eluted and expanded into a T75 flask. G418 selection was maintained at 200 μg/ml, and then cells were passaged and seeded onto coverslips to assess AQP4 expression. NMO rAbs were assayed for binding as described below.

##### Immunostaining

Cells on coverslips were blocked in 10% normal goat serum (NGS)/PBS for 30 min at room temperature. Individual AQP4-positive rAbs at 10–20 μg/ml in 5% NGS/PBS were then applied to coverslips and incubated overnight at 4 °C. The concentration of Ab represented concentrations at or below their apparent *K_d_* values. A secondary control M23-transfected coverslip that was used to correct for background staining of the Alexa Fluor 488 anti-human secondary antibody alone was included and remained in blocking buffer overnight at 4 °C. Coverslips were washed four times in 1× PBS for 3–5 min each, fixed for 5 min with 4% paraformaldehyde, and then blocked in 5% NGS/PBS containing 0.1% Triton X-100. All coverslips were then incubated with 4 μg/ml of a rabbit polyclonal anti-human AQP4 Ab (Santa Cruz Biotechnology) in 2% NGS/PBS for 1 h at room temperature to normalize for transfection efficiency. Coverslips were washed and incubated with 1:600–1:1000 dilutions of both a goat anti-human Alexa Fluor 488 and goat anti-rabbit Alexa Fluor 594 Ab in 2% NGS/PBS for 1 h at room temperature. Cells were washed five times with 1× PBS for 3–5 min each and mounted onto clean microscope slides using Vectashield HardSet Mounting Medium with DAPI (Vector Laboratories).

##### Image Analysis

Immunostained coverslips were visualized on a Nikon E800 fluorescence microscope or on an Olympus IX81 confocal fluorescence microscope using either ×20 or ×40 objective lens. Images were acquired from 4 to 8 nonoverlapping visual fields per coverslip, and images were processed using AxioVision 4.8 software. For studies of Ab binding to AQP4 extracellular loop mutations, 2–5 independently transfected coverslips and 10–30 images were processed for quantitative analyses. The binding intensities of individual NMO rAbs (green fluorescence) and the C-terminal specific positive control anti-AQP4 rabbit polyclonal Ab (red fluorescence) were measured using ImageJ software, and the average background binding of anti-human secondary Ab (green fluorescence) alone to coverslips was subtracted, which typically represented 0.1–3% of rAb binding levels. The average ratio of NMO rAb to control polyclonal Ab (green/red) was calculated for each rAb and wildtype M23 AQP4 to normalize for AQP4 expression and the ratio of the fraction antibody bound on mutant and wildtype M23 AQP4 was then computed and reported as % M23 binding ± S.E. using Equation 1,

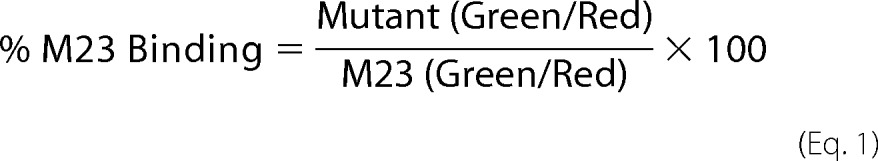


##### Statistical Analysis

The statistical effect of each mutation on binding to M23AQP4 was determined using an unpaired two-sided Student's *t* test with unequal variances.

##### BN-PAGE, SDS-PAGE, and TIRF Microscopy (TIRFM)

**FIGURE 1.**
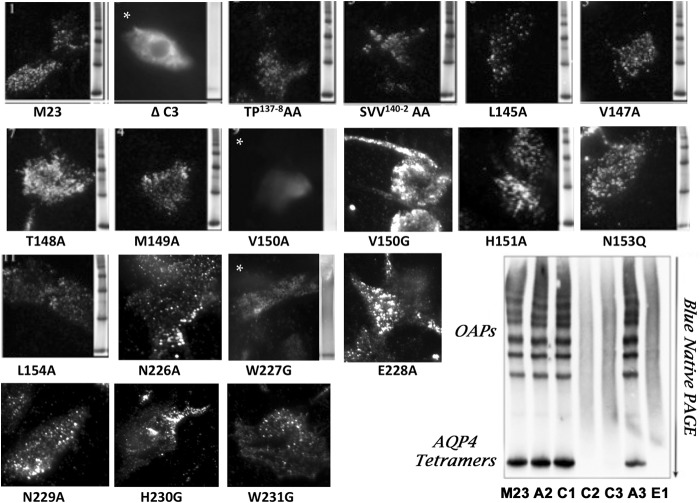
**TIRF microscopy was used to assess the effects of AQP4M23 extracellular loop mutations on surface expression and formation of OAPs following immunostaining with polyclonal control anti-AQP4 antibodies.** For some images, the formation of supramolecular OAPs was further visualized as distinct tetramers and higher order arrays by BN-PAGE. Images with a *white asterisk* indicate mutations impairing the formation of OAPs as judged by both TIRF microscopy and BN-PAGE. *Lower right panel,* BN-PAGE showing the effects of extracellular serial loop mutations on OAP formation.

Transfected U-87MG cells were collected 24–48 h post-transfection, washed in cold PBS, and lysed in native PAGE sample buffer (Invitrogen) containing 0.5% dodecyl-β-d-maltose. Samples were then electrophoresed through blue native gels, transferred onto PVDF membrane, and probed with a polyclonal anti-rabbit antibody against the intracellular C terminus of AQP4 Ab as described (10). Formation of OAPs was visualized by the characteristic ladder appearance (Fig. 1). Alternatively, OAP formation in transfected cells was visualized for some mutations by TIRF microscopy revealing a characteristic punctate staining at the cell surface (Fig. 1). The effects of serial and point mutations on tetramer and OAP formation are reported for each construct in Table 1.

For SDS-PAGE analysis of NMO rAb binding to AQP4 M23, low speed cell lysates were prepared from AQP4-transfected cells and quantified by the BCA protein assay (Pierce), and 20 μg of protein were resolved on a SDS-12.5% polyacrylamide gel. Proteins were electrophoretically transferred to nitrocellulose, dissected into individual lanes, blocked in 3% BSA, and then incubated with 1–2 μg/ml of the indicated NMO CSF-derived human rAb or the polyclonal anti-rabbit intracellular AQP4 Ab. Nitrocellulose strips were then washed in TBS containing 0.1% Tween 20 (TBS/Tween 20), incubated with goat anti-human HRP- or goat anti-rabbit HRP-conjugated secondary antibody in 3% BSA, and then rewashed in TBS/Tween 20. Binding was visualized by chemiluminescence (Supersignal West Pico kit, Pierce).

##### Mapping of M23-AQP4-binding Sites

The three-dimensional mapping of putative AQP4 epitopes was based on the crystal structure of human M1-AQP4 (Protein Data Bank code 3DG8) at 1.8 Å (16). Three-dimensional images of AQP4 monomers and tetramers were obtained and manipulated using Jmol Viewer at the Protein Data Bank. False colorations of AQP4 extracellular domains were performed manually on exported images using Adobe Photoshop.

## RESULTS

### 

#### 

##### Monoclonal Anti-AQP4 NMO Antibodies Do Not Recognize Linear Epitopes on AQP4

**FIGURE 2.**
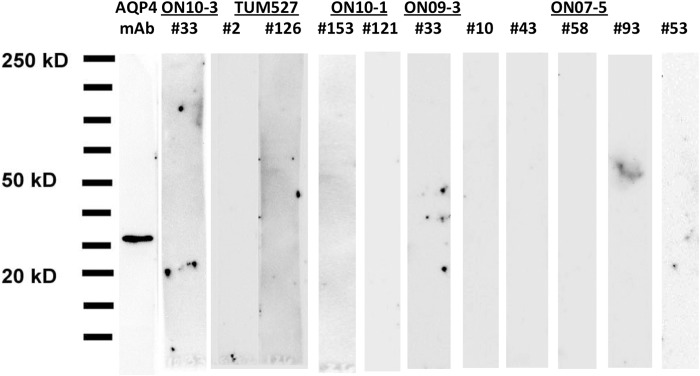
**SDS-PAGE immunoblotting was used to determine binding of NMO CSF-derived AQP4-specific rAbs to a CHOK1 cell protein extract following transfection with the M1 isoform of human AQP4.** A mouse anti-AQP4 mAb was used as positive control and shows binding to a single ∼26-kDa protein. None of 12 individual AQP4-specific rAbs recognized human AQP4 under denaturing conditions.

FACS single-cell sorting was used to isolate single CD138^+^ antibody-secreting cells from the CSF of five NMO-IgG-positive patients. Sequencing of their PCR-amplified heavy- and light-chain variable regions identified clonal populations within each repertoire, and Abs were generated from each clone as described previously (3). NMO CSF-derived rAbs specific for AQP4 failed to recognize denatured AQP4 protein monomers in SDS-PAGE protein immunoblots of lysates from AQP4-transfected CHOK1 cells (Fig. 2), indicating that CSF-derived Abs likely recognize conformational epitopes formed by the assembly of AQP4 tetramers and OAPs at the cell surface.

##### Mutagenesis of AQP4 Extracellular Domains Identifies Two Patterns of AQP4 Recognition

**FIGURE 3.**
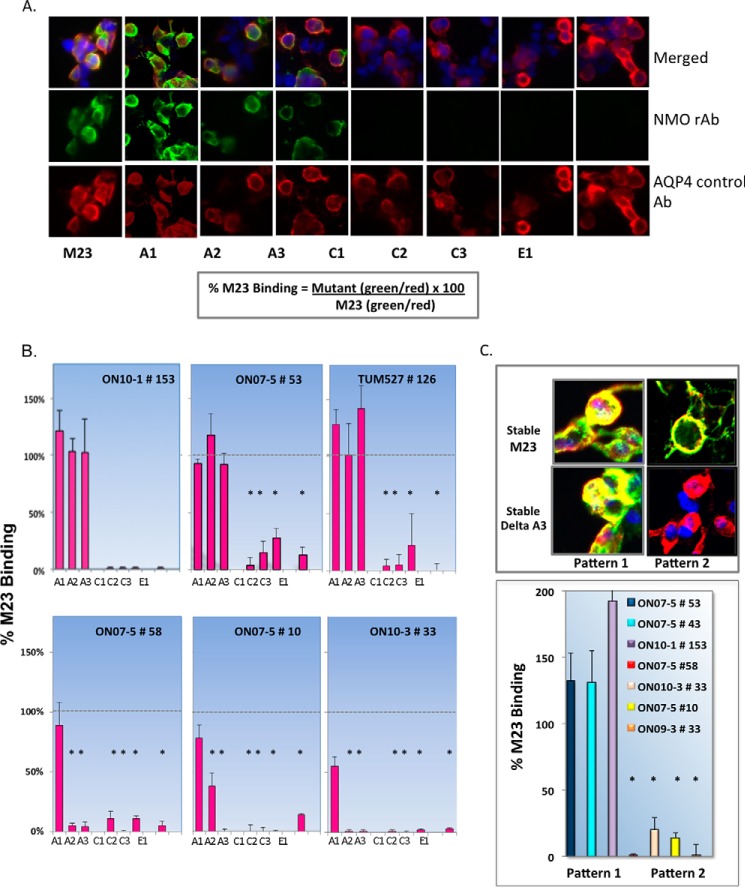
**Serial mutations engineered into each extracellular loop of the M23 isoform of human AQP4 reveal two patterns of antigen recognition by NMO CSF-derived rAbs.** *A,* double immunofluorescent staining with AQP4-specific NMO rAbs (*green channel*) and polyclonal anti-AQP4 control Abs (*red channel*) was used to quantify the effects of serial loops A, C, and E mutations on AQP4 cell binding using Equation 1 (see under “Experimental Procedures”). Each AQP4 serial mutation is described in Table 1. *B,* binding of six NMO rAbs to serial AQP4 mutations identifies two patterns of AQP4 recognition that are distinguished by their sensitivity to loop A substitutions ΔA2 and ΔA3 (Table 1). Bar values represent the mean value of multiple images ± S.E. *, *p* < 0.01, *t* test. *C,* stable U-87MG cell lines expressing M23 or the serial AQP4 mutation ΔA3 were produced and assayed to confirm dependence of pattern 2 NMO rAbs on loop A sequences. *Top panel* shows the merged images of representative pattern 1 (loop A-independent) and pattern 2 (loop A-dependent) NMO rAb binding to M23 and ΔA3 stable cell lines. *Bottom panel* quantifies the binding differences of pattern 1 and pattern 2 NMO rAbs to cells stably expressing the ΔA3 mutation (*, *p* < 0.01, *t* test).

To define regions of the AQP4 extracellular domain important for Ab recognition, we first introduced a series of alanine and/or glycine substitutions into the three extracellular domains of human M23 AQP4 as listed in Table 1. This included three sets of nonoverlapping serial substitutions covering loop A (ΔA1-ΔA3) and loop C (ΔC1-ΔC3), and one serial substitution that covers loop E (ΔE1). Plasmid DNA of each mutation and M23 AQP4 were separately transfected into U-87MG glioblastoma or HEK-EBNA cell lines, and binding to AQP4 was assayed by a dual immunostaining protocol in which AQP4 was immunostained using a polyclonal Ab recognizing the intracellular AQP4 C terminus. The double staining of normal and mutated AQP4-transfected cells with ONO7-5 #53 rAb (green fluorescence) and the AQP4-positive control Ab (red fluorescence) are shown in Fig. 3*A*. Whereas binding of the positive control Ab is largely unaltered in cells expressing AQP4 serial extracellular loop substitutions, binding of rAb #53 is absent in cells expressing the ΔC1-ΔC3 and ΔE1 mutations. Other NMO rAbs displayed additional sensitivity to the serial substitutions ΔA2 and ΔA3 in loop A (Fig. 3*B*). As summarized in Table 2 for all AQP4-specific rAbs studied, both pattern 1 (loop A-independent) and pattern 2 rAbs (loop A-dependent) were recovered from each NMO patient. Using a U-87MG stable cell line expressing the ΔA3 AQP4 substitution mutation, we confirmed that all pattern 2 rAbs required intact extracellular loop A sequences (Fig. 3*C*). The necessity of individual regions within loops C and E (Table 1) remained unclear as the serial substitutions ΔC2, ΔC3, and ΔE1 eliminated plasma membrane expression and OAP formation as measured by BN-PAGE and TIRFM (see Fig. 1). Because of the uninformative nature of these large serial substitutions, point mutations that did not disrupt AQP4 plasma membrane expression and OAP assembly were required to generate a detailed map of individual amino acids contributing to epitope formation.

**TABLE 1:** **Mutations introduced into the extracellular domains of AQP4**

AQP4 mutation*^a^*	Mutational changes*^a^*	Mutated amino acid positions*^b^*	Tetramer and OAP array formation*^c^*
**Loop A**			
ΔA1	TINW AAAG	56–59	OAP
ΔA2	TEKP AAGA	62–65	OAP
ΔA3	LPVD AAAA	66–69	OAP
T62A	AEKP AEKP	62	OAP
E63A	TEKP TAKP	63	OAP
K64A	TEAP TEAP	64	OAP
P65A	TEKA TEKA	65	OAP
L66A	LPVD APVD	66	OAP
P67A	LPVD LAVD	67	OAP
V68A	LPVD LPAD	68	OAP
D69A	LPVD LPVA	69	OAP

**Loop C**			
ΔC1	TPPSVV AAAGAA	137–142	OAP
ΔC2	LGVTM AGAAA	145–149	NEG
ΔC3	VHGNLT AAGAAA	150–155	NEG
TP137–8AA	TPPSVV AAPSVV	137–138	OAP
SVV140–2AAA	TPPSVV TPPAAA	140–142	OAP
S140A	TPPSVV TPPAVV	140	OAP
V141A	TPPSVV TPPSAV	141	OAP
V142A	TPPSVV TPPSVA	142	OAP
L145A	LGVTM AGVTM	145	OAP
V147A	LGVTM LGATM	147	OAP
T148A	LGVTM LGVAM	148	OAP
M149A	LGVTM LGVTA	149	OAP
V150A	VHGNLT AHGNLT	150	NEG
V150G	VHGNLT GHGNLT	150	OAP
H151A	VHGNLT VAGNLT	151	OAP
N153Q	VHGNLT VHGQLT	153	OAP
N153A	VHGNLT VHGALT	153	OAP
L154A	VHGNLT VHGNAT	154	OAP
T155A	VHGNLT VHGNLA	155	OAP

**Loop E**			
ΔE1	NWENHW AGAAAG	226–231	NEG
N226A	NWENHW AWENHW	226	OAP
W227G	NWENHW NGENHW	227	NEG
E228A	NWENHW NWANHW	228	OAP
N229A	NWENHW NWEAHW	229	OAP
H230G	NWENHW NWENGW	230	OAP
W231G	NWENHW NWENHG	231	OAP

*^a^* The indicated amino acids located within the A, C, and E extracellular domains of the M23 isoform of human aquaporin 4 were mutated to alanine and or glycine residues as shown in column 2. An additional asparagine to glutamine mutation was also introduced at position 153.

*^b^* Amino acids are numbered according to the M1 isoform of hAQP4 as published by Ho *et al.* (16).

*^c^* The effects of amino acid mutations on the formation of tetramers and OAPs were assessed by BN-PAGE and by TIRF immunostaining (see Fig. 1).

**TABLE 2:** **NMO rAb binding to serial amino acid substitutions in AQP4 extracellular loops** Quantification of rAbs derived from five different NMO patients to cells transiently expressing the M23 isoform of human AQP4 and to cells expressing M23 AQP4 mutated in the indicated extracellular domain as described. Values are reported as percent of rAb binding ± S.E. observed to the nonmutated M23 human AQP4 isoform. Values indicated in boldface represent statistically significant differences in binding to the indicated loop mutation using an unpaired two-sided *t* test with unequal variances. *, *p* < 0.01.

NMO rAb		Loop A (% M23 binding)			Loop C (% M23 binding)			Loop E (% M23 binding), ΔE1
		ΔA1	ΔA2	ΔA3	ΔC1	ΔC2	ΔC3	
**Pattern 1**								
ON10-3	18	116 ± 12	170 ± 23	63 ± 10	0.1 ± 0.1*	0.2 ± 0.3*	1.2 ± 0.7*	0.1 ± 0.4*
ON07-5	43*^a^*	100 ± 14	162 ± 22	156 ± 38	**0 ± 10**	**0 ± 9**	**0 ± 9**	**0 ± 9**
	53	93 ± 4	118 ± 19	92 ± 10	4 ± 7*	15 ± 10*	28 ± 8*	13 ± 7*
ON10-1	153*^a^*	121 ± 19	103 ± 12	102 ± 30	1 ± 1*	1 ± 1*	1 ± 1*	1 ± 1*
TUM527	126	128 ± 13	101 ± 28	142 ± 20	4 ± 6*	5 ± 9*	22 ± 28*	0 ± 6*
	2*^a^*	111 ± 8	134 ± 11	90 ± 14	36 ± 7*	20 ± 4*	23 ± 5*	13 ± 4*
	68*^a^*	ND*^b^*	122 ± 17	121 ± 12	6 ± 10*	6 ± 4*	8 ± 7*	22 ± 5*

**Pattern 2**								
ON10-3	33	55 ± 8	**1 ± 1**	1 ± 1*	1 ± 1*	0 ± 1*	2 ± 0.2*	3 ± 0.1*
ON07-5	10	78 ± 11	38 ± 11*	0 ± 2*	0 ± 5*	0 ± 3*	0 ± 1*	14 ± 1*
	58*^a^*	89 ± 19	5 ± 2*	4 ± 4*	11 ± 6*	0 ± 1*	11 ± 2*	5 ± 4*
ON10-1	121	130 ± 9	0 ± 28*	11 ± 5*	0 ± 2*	0 ± 1*	0 ± 1**	4 ± 7*
ON09-3	33*^a^*	55 ± 8	0 ± 0.5*	0 ± 0.2*	1 ± 0.3*	0 ± 0.3*	2 ± 0.2*	3 ± 1*

*^a^* AQP4 loop mutations were expressed in brain tumor-derived U-87MG cells. Comparable results were observed for rAb 58 binding to HEK293 cells expressing AQP4 loop mutations.

*^b^* ND means not done.

##### Single Amino Acid Substitutions Identify Critical Residues Required for AQP4 Recognition

We next introduced alanine and or glycine replacement mutations for most amino acid in loops A, C, and E, excluding native glycine residues (Table 1). To identify specific amino acids within each loop contributing to AQP4 recognition, we assayed three pattern 2 NMO rAbs for binding to each of the loop A mutations and assayed three pattern 1 and three pattern 2 NMO rAbs for binding to each loop C and E mutation. The point mutation W227G in loop E eliminated AQP4 plasma membrane expression and OAP formation preventing any assessment of the role of this amino acid in pattern 1 or pattern 2 rAb binding. Because the ΔA1 serial substitution did not alter binding of any AQP4-specific rAbs, point mutations were not generated in this region of loop A.

**FIGURE 4.**
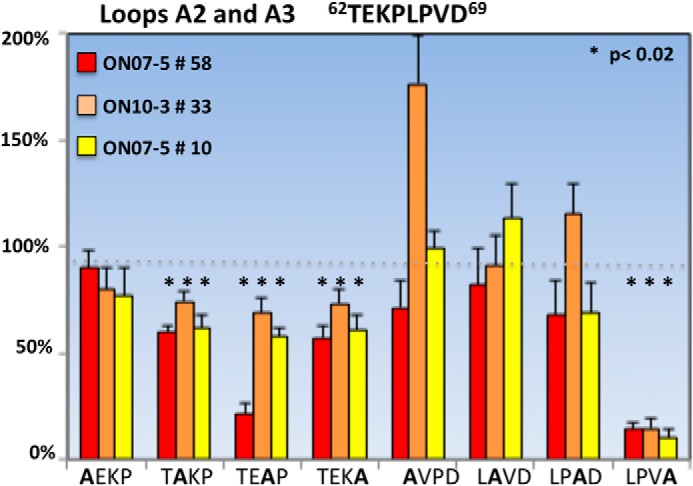
**Single amino acid substitutions identify loop A amino acids important for pattern 2 NMO rAb binding to AQP4.** Single point mutations in the four amino acid regions ^62^TEKP^65^ and ^66^LPVD^69^ significantly reduce the binding of the indicated pattern 2 rAbs to M23 AQP4. Values are reported as % M23 binding ± S.E. (*, *p* < 0.01, *t* test). Single mutations were not introduced into region A1.

Single alanine substitutions within loop A sequence ^62^TEKP^65^ identified the amino acids EKP at positions 63–65 as important contributors to pattern 2 Ab binding (Fig. 4). A modest but significant reduction in binding was observed at each of these positions suggesting that this trio of amino acids together help facilitate binding of pattern 2 rAbs to AQP4. ON07-5 #58, a human AQP4-specific rAb, was most affected by the K64A point mutation, which also represents a species-specific difference between human and mouse AQP4. In the region (^66^LPVD^69^) covered by the loop A serial substitution ΔA3, the only residue impacting AQP4 binding was Asp^69^, which produced a significant loss of binding for all pattern 2 rAbs (Fig. 4). Pisani *et al.* (14) recently reported that a D69H substitution affected loop A conformation and serum NMO-IgG binding.

**FIGURE 5.**
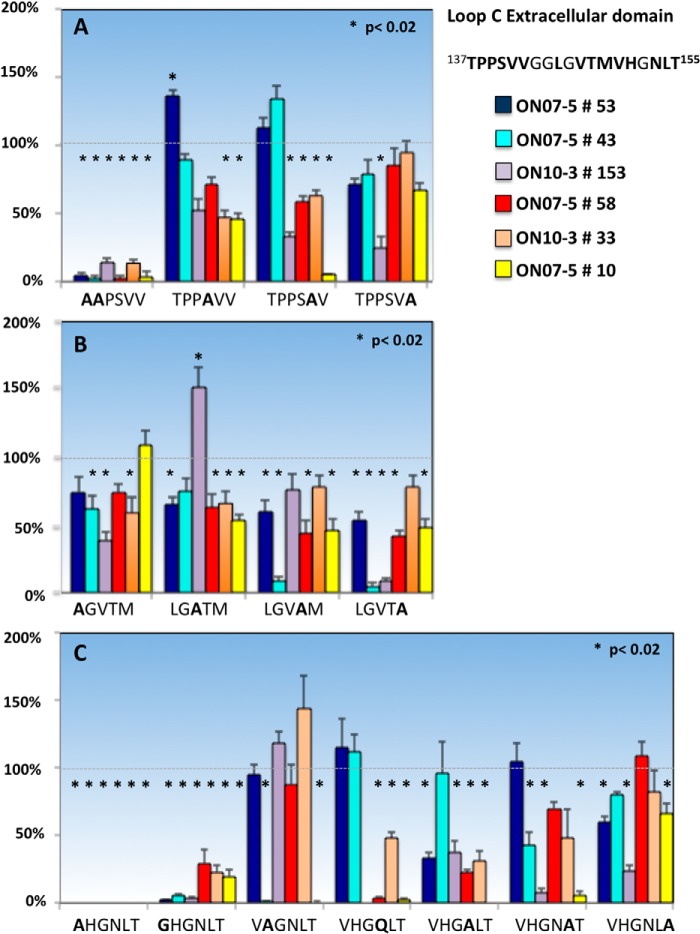
**Single alanine or glycine amino acid substitutions identify loop C amino acids contributing to NMO rAb binding to AQP4.** Multiple point mutations and one double point mutation in loop C significantly reduce patterns 1 and 2 AQP4 rAb binding. None of the four glycines in loop C were altered. The amino acid Val^150^ was mutated to both a glycine and alanine because of the differential effects on tetramer and OAP formation. Asn^153^ was mutated to both an alanine and a conserved glutamine. Values are reported as % M23 binding ± S.E. (*, *p* < 0.02, *t* test). Experiments measuring binding of ON07-5 #10 rAb to cells expressing the N153A mutation and binding of ON10-1 #153 rAb to cells expressing the N153Q are not included in this analysis.

Loop C is the largest AQP4 extracellular domain and appears critical for rAb recognition as judged by the absence of rAb binding to M23 AQP4 mutant ΔC1. The crystal structure of human AQP4 reveals an α-helix at positions ^139^PSVV^142^ within the N-terminal portion of loop C (16). To determine whether this helix might be important for antigen binding, we first generated two smaller point mutations (TP137–8AA and SVV140–2AAA). The TP137–8AA mutation greatly reduced the binding of every AQP4-specific rAb assayed (Fig. 5). The effects of the SVV140–2AAA mutation were mixed and varied by individual rAbs independent of their pattern 1 or pattern 2 classifications (data not shown). Single point mutations introduced at positions Ser^140^, Val^141^, and Val^142^ and across the region ^145^LGVTM^149^ further illustrated these Ab-specific positional effects, which ranged from modestly increased binding to AQP4 for some pattern 1 Abs (ON07-5 #53 at Val^141^ and ON10-1 #153 at Val^147^) to moderately decreased binding for many other rAbs. Mutations causing the greatest disruption of individual rAb binding within this region included V142A for ON07-5 #10, T148A and M149A for ON07-5 #43, and M149A for ON10-1 #153. The Met^149^ amino acid is specific to human AQP4 reflecting the specificity of ON07-5 #43 Ab for human AQP4. The species specificity of ON10-1 #153, which was also significantly affected by this mutation, has not been determined. Experiments to independently evaluate the contributions of Thr^139^, Pro^140^, and Pro^141^ were not performed. Excluding the ^137^TP^138^ amino acids, which were necessary for the binding of all NMO rAbs, no other single mutation encompassed within the ΔC1 and ΔC2 mutations unambiguously distinguished pattern 1 from pattern 2 binding.

Point mutations at Val^150^ in loop C identified this amino acid as critical for AQP4 recognition by all NMO rAbs. When alanine was substituted for Val^150^, plasma membrane expression and OAP formation were abrogated thus preventing NMO rAb binding (Figs. 1 and 5*C*); however, a V150G substitution at this position maintained OAP formation, but significantly reduced binding for each of the six NMO rAbs assayed, with pattern 1 Abs having a somewhat greater sensitivity than pattern 2 rAbs. A second critical amino acid within the C-terminal portion of loop C that influenced the binding of both pattern 1 and 2 rAbs was Asn^153^, which is part of a consensus *N*-glycosylation motif (^153^NLT^155^). A conservative N153Q substitution abolished or significantly reduced the binding of pattern 2 rAbs but not pattern 1 rAbs, whereas a second N153A substitution reduced the binding of all pattern 1 and 2 rAbs with the exception of ON07-5 #43, which was not affected by either substitution. The T155A mutation did not produce equivalent global reductions in pattern 2 binding indicating that the entire consensus sequence was not required.

**FIGURE 6.**
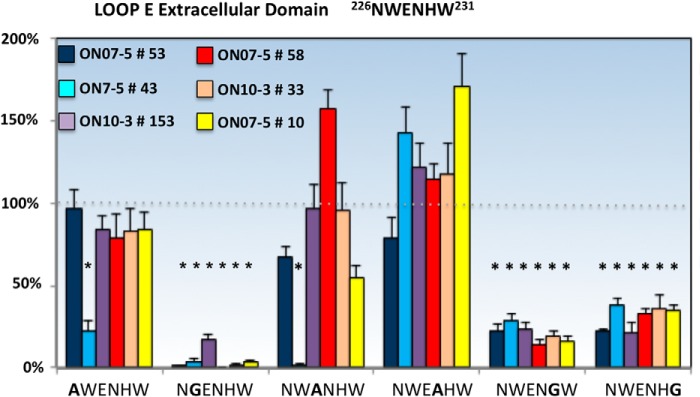
**Single amino acid substitutions identify loop E amino acids important for NMO rAb binding to AQP4.** Three loop E point mutations significantly reduce the binding of all NMO rAbs to M23 AQP4. rAb ON07-5 #43 is uniquely sensitive to the point mutation at Glu^228^. Values are reported as % M23 binding ± S.E. (*, *p* < 0.01, *t* test).

Loop E (^226^NWENHW^231^) mutations revealed three residues (Trp^227^, His^230^, and Trp^231^) that significantly affected the binding of each NMO rAb (Fig. 6). Unfortunately, the W227A substitution was uninformative as it abolished AQP4 plasma membrane and OAP formation (Table 1). The single point mutations H230G and W231G reduced the binding of all NMO rAbs without impacting OAP formation. ON07-5 #43 Ab represented a unique pattern 1 rAb with additional sensitivities to mutations N226A and E228A.

##### Loop C Amino Acids His^151^ and Leu^154^ Define a Subset of Pattern 2 Binding

**FIGURE 7.**
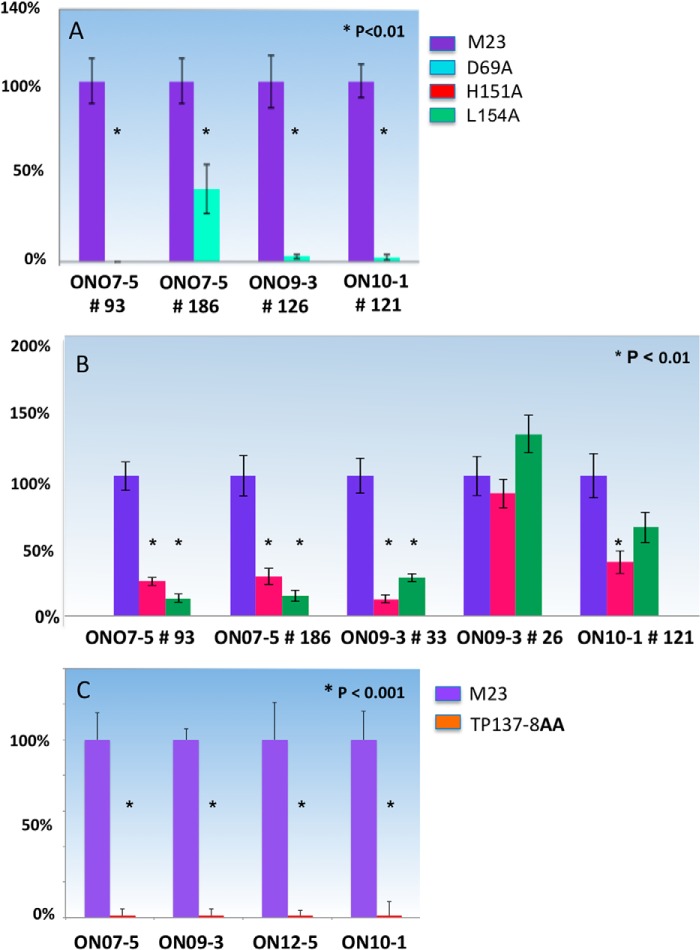
**Pattern 2 rAbs dependent on the loop C amino acids His^151^ and Leu^154^ further define a unique subset of pattern 2 NMO rAbs.** *A,* binding of pattern 2 AQP4-specific rAbs are sensitive to the loop A point mutation D69A. *B,* subset of pattern 2 rAbs are uniquely dependent on His^151^ and Leu^154^ for AQP4 recognition. *C,* loop C mutation TP137–8AA abrogates cell binding of serum NMO-IgG to M23 AQP4. The indicated sera (1:20 dilution) from four NMO patients were assayed for binding to U-87MG cells expressing either M23 or TP137–8AA AQP4. Values are reported as % M23 binding ± S.E. (*, *p* < 0.001, *t* test).

The H151A and L154A mutations identify critical loop C amino acids that further delineate the heterogeneity of AQP4 recognition by AQP4-specific rAbs. Binding of both the pattern 1 rAb, ON07-5 #43, and pattern 2 rAb, ON07-5 #10, was severely impaired by the H151A substitution. ON07-5 #10 binding was also significantly reduced by the L154A substitution as was the pattern 1 rAb ON10-1 #153, and to a somewhat lesser extent, ON07-5 #43. To determine whether this sensitivity defines pattern subsets, additional rAbs were first screened for binding to cells expressing the stable ΔA3 mutation to determine their dependence on loop A (Fig. 7*A*), followed by binding assays that determined their sensitivity to the H151A and L154A mutations (Fig. 7*B*). Additional pattern 2 rAbs (ON07-5 #93, ON09-3 #33, and ON07-5 #186) showed a clear dependence on the presence of His^151^ and Leu^154^, whereas a fourth pattern 2 rAb, ON10-1 #121 was only partially dependent.

##### C Loop Mutations Abolish Binding of NMO Patient Sera

To assess whether amino acids necessary for AQP4 rAb binding were critical for binding of NMO sera, sera from four patients in this study were tested for binding to mutant AQP4 TP137–8AA (Fig. 5*C*). As observed for all NMO rAbs, the TP137–8AA mutation abolished the binding of each NMO-IgG to M23 AQP4 indicating the global importance of loop C conformation to antigen recognition.

## DISCUSSION

The binding of NMO patient-derived antibodies to the extracellular domains of AQP4 initiate an immune-mediated pathology that is characterized by activation of complement, astrocyte cytotoxicity, inflammation with granulocyte infiltration, and oligodendrocyte cell death leading ultimately to demyelination (3, 6, 17, 18). Although first described as a serum response, plasmablasts secreting anti-AQP4 Abs are clonally expanded within the CSF compartment and may be a significant source of AQP4-specific CNS Abs (3, 19). To study the CSF Ab response in NMO, we have used single-cell PCR to generate a panel of AQP4-specific monoclonal rAbs from expanded clones of plasmablasts present in CSF of NMO patients. The rAb products of these NMO patient-derived plasmablasts have reproduced CNS tissue pathology in multiple models of NMO lesion pathogenesis (3, 5, 20) demonstrating their ability to reproduce the pathogenicity of serum NMO-IgG. Herein, we used a panel of 12 CSF-derived AQP4-specific Abs from five NMO-IgG seropositive patients to evaluate the importance of each amino acid in the three extracellular loops of AQP4 for NMO-IgG recognition. To assess binding in its native state, cells were transfected with one of a series of 37 mutated AQP4 M23 constructs, and their binding was quantified by fluorescence microscopy with normalization for AQP4 expression using a second Ab that detects a linear intracellular AQP4 peptide.

In contrast to prior studies using polyclonal human serum containing a broad collection of antibodies with a wide range of affinities and epitopes, our use of AQP4-specific monoclonal rAbs allowed us to determine distinct amino acids important for the binding of different classes of AQP4 autoantibodies. A common feature of each NMO rAb was the importance of conformation and quaternary structure for AQP4 recognition. All CNS-derived AQP4-specific rAbs examined to date do not recognize AQP4 protein in denaturing SDS-PAGE immunoblots but require the expression of conformationally intact protein on the plasma membrane. In this study, all serial or single amino acid substitutions within AQP4 extracellular domains that abrogated M23 plasma membrane expression and OAP formation (Table 1) abolished AQP4 rAb binding relative to that of the C-terminal specific polyclonal control Ab. This included serial substitutions ΔC2, ΔC3, and ΔE1 as well as the single point mutations V150A in loop C and W227G in loop E. We hypothesize that these mutations are likely impairing AQP4 tetramer oligomerization and/or transport of AQP4 tetramers from the endoplasmic reticulum to the cell surface based on TIRFM (Fig. 1). Interestingly, although the ΔC2 mutant prevented plasma membrane expression and OAP formation, none of the four constituent amino acids (LVTM) produce the same inhibition when mutated individually. Together, these observations indicate the presence of important topology within the AQP4 extracellular domains that is necessary for oligomerization and/or transport to the cell surface.

While conformational membrane-expressed AQP4 was essential for NMO rAb binding, prior studies have reported serum NMO-IgG binding to AQP4 peptides, monomers, and higher order arrays (13, 19, 21). The distinction is due to the polyclonal nature of serum NMO-IgG, which contains many AQP4 autoantibodies directed against multiple targets as follows: conformational AQP4 protein, denatured AQP4 multimers and monomers, and AQP4 peptides. The relevance of AQP4 autoantibodies directed against denatured AQP4 or AQP4 peptides in disease pathogenesis, however, remains unclear. Although NMO CSF rAbs have been shown to bind to AQP4-expressing cells or CNS astrocytes and mediate target destruction by *in vitro*, *ex vivo*, and *in vivo* methods (3, 5, 20), the ability of autoantibodies against denatured or linear AQP4 epitopes to bind plasma membrane AQP4 and activate antibody effector function has not been tested. In one study, a significant proportion of anti-AQP4 antibodies targeted linear epitopes localized in the intracellular domains of the protein (21), and in a second study, serum antibodies against loops A and E peptides lacked disease specificity (13). Although serum Abs to denatured linear AQP4 epitopes may be diagnostic for seropositive NMO (13), the uniform absence of such broad specificity to denatured epitopes in AQP4-specific rAbs derived from NMO CSF implies distinctions between global anti-AQP4 responses and the AQP4-specific B cells infiltrating the CNS. Pathogenicity is likely driven by conformationally dependent Abs binding to surface-exposed epitopes in tetramers that are stabilized or optimized in OAPs (10).

**FIGURE 8.**
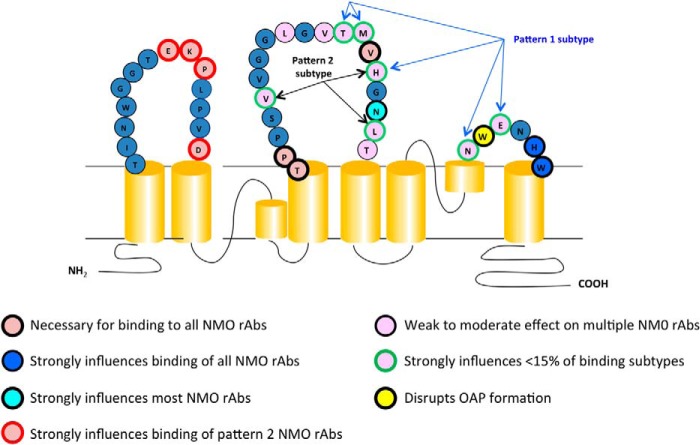
**Schematic of AQP4 shows the extracellular loop amino acids contributing to NMO rAb binding patterns.**

Our analysis revealed amino acids that were either critical for the binding of all AQP4-specific rAbs or defined unique epitopes specific to certain rAbs. Five AQP4 constructs greatly impaired NMO rAb recognition as follows: C loop mutants TP137–8AA and V150G (Fig. 8, *purple*), C loop mutant N153A (Fig. 8, *turquoise*), and E loop mutants H230G and W231G (Fig. 8, *blue*). The most remarkable feature distinguishing patterns of AQP4 antigen recognition is their differential sensitivity to mutations within the first extracellular domain, broadly categorized as either pattern 1 (loops C- and E-dependent) or pattern 2 (loops A-, C, and E-dependent) binding. Importantly, all the pattern 2 rAbs appear to be sensitive to mutagenesis of the same amino acids within the loop A domain (Fig. 8, highlighted in *red*). All pattern 2 rAbs are uniquely sensitive to both the ΔA2 and ΔA3 serial substitutions (Table 2 and Fig. 3*D*) and single amino acid mutations at Asp^69^ and the sequence ^63^EKP^65^. Although most pattern 1 and pattern 2 rAbs share the same sensitivity to the loop C mutants TP137–8AA, V150G, and N153A, a subset of pattern 2 rAbs, derived from different patients (Figs. 7 and 8) showed additional dependence on loop C amino acids His^151^ and Leu^154^. This sub-pattern may be further defined by the amino acid Val^141^, based on ON07-5 #10 rAb staining, but it has not been verified with other pattern 2 rAbs dependent on His^151^ and Leu^154^ (Fig. 8). There are also multiple amino acids across loop C that have modest effects on the binding of individual rAbs, independent of their pattern classification, but do not delineate clear AQP4 epitopes, suggesting that subtle changes to the overall conformation of AQP4 extracellular domains influence AQP4-IgG binding. The epitopes of Abs classified as pattern 1 are less well defined than pattern 2 Abs and show modest to strong sensitivities to unique amino acid substitutions across both loops C and E. The most unique pattern 1 Ab is ON07-5 #43, which displays a specific requirement for amino acids Thr^148^, Met^149^, His^151^, Asn^226^, and Glu^228^, and is the only rAb studied that is not affected by the N153A substitution.

**FIGURE 9.**
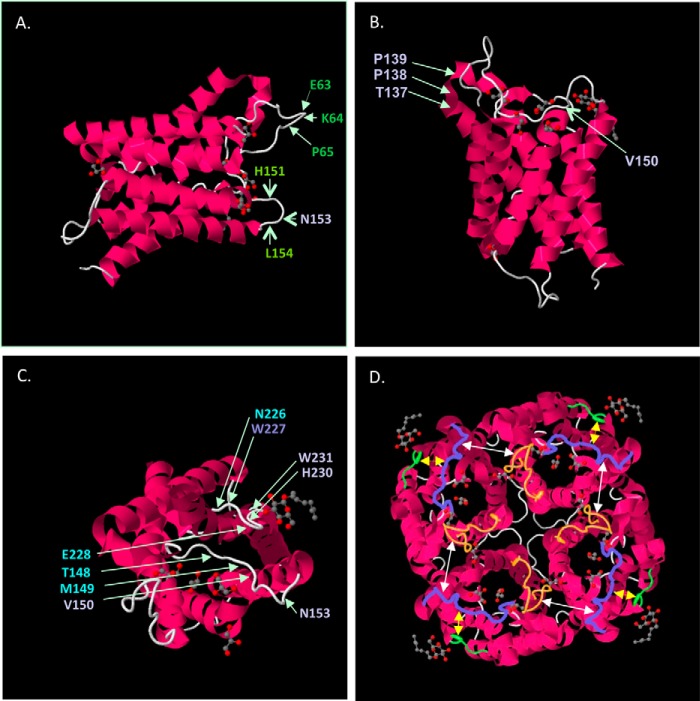
**Extracellular amino acids critical for AQP4 binding are closely positioned in the three-dimensional structure of AQP4.** *A,* right-handed lateral view of an AQP4 monomer shows the projection from the cell surface of key amino acids contributing to pattern 1 and pattern 2 binding. *B,* top view of an AQP4 monomer tilted toward the viewer shows critical amino acids identified by mutagenesis that contribute to loop C conformation. *C,* front view of an AQP4 monomer shows a putative binding pocket formed by parallel regions of loop C and loop E. The locations of critical amino acids identified by point mutations are *highlighted. D,* top view of an AQP4 tetramer reveals the juxtaposition of critical amino acids within and between monomers contributing to both pattern 1 and pattern 2 binding. *White arrows* show the alignment and distance between loop A and loop C on adjacent AQP4 monomers. *Yellow arrows* show the distance between adjacent loop C and loop E sequences within monomers. The extracellular domains are false-colored: loop A, *gold*; loop C, *purple*; and loop E, *green*.

The present analysis is limited by the inability to differentiate mutations that directly affect Ab contact from those that cause both global and local conformational changes. To preliminarily address this issue, we examined the three-dimensional structure of human AQP4 to investigate the juxtaposition of critical amino acids that likely define AQP4 epitope domains. Fig. 9 displays the crystal structure of human M1 AQP4 at 1.8 Å resolution (16) visualized at different angles to show the position of critical amino acids defined by this mutagenesis study. Because OAP formation drives high affinity binding of most CSF-derived rAbs and are formed from the M23 AQP4 isoform (10), a detailed examination of putative rAb-AQP4 interactions across OAPs is not feasible. Nevertheless, the following features are notable when viewing the three-dimensional structure of M1 AQP4 tetramers. First, amino acids projecting farthest outward from the extracellular surface form local structures comprised of critical residues required for NMO rAb binding (Fig. 9*A*). They include the loop A amino acids ^63^EKP^66^ that are critical for all pattern 2 rAb binding, and the loop C amino acid Asn^153^ that is located on the surface of a horseshoe bend. This prominent bend within loop C also includes His^151^ and Leu^154^ that delineate a subset of pattern 2 Abs. Gly^152^ is also located within this bend, but its putative contribution to AQP4 recognition has not been investigated. Second, additional amino acids critical for NMO rAb binding are positioned in regions that likely dictate the correct three-dimensional spacing and conformation of extracellular loops (Fig. 9*B*). The loop C amino acids ^137^TPP^139^ create an almost 180° turn that positions loop C parallel to much of the extracellular surface of AQP4, whereas the other critical loop C amino acid Val^150^ creates an angle that directs the prominent loop C horseshoe (^151^HGNL^154^) perpendicular from the plane of the cell surface. The role of Asp^69^ in pattern 2 binding is not readily apparent from the three-dimensional models, but it has been reported to affect loop A conformation (14). Third, although loop E does not protrude outward from the cell surface as far as loops A and C, it runs parallel to a portion of loop C (^145^LGVTM^149^) forming a pocket that may be necessary for both pattern 1 and pattern 2 binding. It may also explain the unique sensitivity of individual rAbs to amino acid mutations in this region (Fig. 7*C*). Loop E also forms a protruding horseshoe bend that brings the loop C amino acids Thr^148^ and Met^149^ in close proximity (∼7–8 Å) to the loop E amino acids Asn^226^, Trp^227^, and Glu^228^, positions that uniquely define the pattern 1 epitope represented by NMO rAb ON07-5 #43. Finally, the three-dimensional structure of the AQP4 tetramer reveals important structural features that likely contribute to epitope recognition. The first and most striking feature is the juxtaposition of loop A amino acids ^63^EKP^65^ in one monomer to the ^151^HGNL^154^ amino acids of loop C in the adjacent monomer (∼16 Å), thus aligning the two prominent extracellular bends in each loop critical to pattern 2 rAb binding (Fig. 9*D*). The second feature is the location of loop E at the periphery of each monomer subunit, possibly providing access for rAb recognition of the pocket created by loops C and E (Fig. 9*D*).

The epitopes identified using NMO rAbs show some similarities to those identified previously using serum NMO-IgG, including the absence of binding to denatured AQP4 by protein immunoblotting and the enhanced staining of AQP4 in cells forming supramolecular OAPs (22). Select mutagenesis of some human AQP4 extracellular amino acids based on their sequence homologies to other OAP-forming aquaporins also revealed two major conformational epitopes that partially overlapped with the critical residues differentiating both pattern 1 (loops C- and E-dependent) and 2 (loops A-, C-, and E-dependent) binding (12); they include the serial mutation of amino acids ^61^GTEK^64^ in loop A, ^146^GVTMV^150^ in loop C, and ^227^WE^228^ in loop E. Using our panel of NMO monoclonal rAbs, we have identified novel amino acids critical for the binding of all (^137^TP^138^ and Val^150^), most (Asn^153^), or subsets (Val^141^, His^151^, and Leu^154^) of AQP4-specific rAbs. Previous studies have revealed consistently greater affinity of NMO-IgG binding to M23 than M1 AQP4 (10), and Fab fragments of NMO rAbs showed a similar preference of AQP4 isoform binding, indicating that structural changes in AQP4 upon array assembly, and not bivalent cross-linking of whole IgG, result in the greater binding affinity to OAPs. Whether AQP4 OAPs create novel surface epitopes or stabilize conformational epitopes on AQP4 tetramers remains to be determined.

In conclusion, NMO CSF-derived AQP4-specific B cells show distinct patterns of antigen recognition that can be classified into two broad categories based on their differential dependence on select loop A amino acids. Localization of the critical amino acids in the AQP4 crystal structure identifies pockets or domains both between and within monomers that align important loop A, loop C, and loop E amino acids and contribute to antigen recognition. These domains may be important for development of potential therapies designed to inhibit NMO-IgG interaction with AQP4 and subsequent CNS lesion formation.

## Footnotes

2 The abbreviations used are:

NMO: neuromyelitis optica
rAb: recombinant monoclonal Ab
AQP4: aquaporin 4
BN: blue native
OAPs: orthogonal array of particles
TIRF: total internal reflection fluorescence
TIRFM: TIRF microscopy
NGS: normal goat serum
Ab: antibody.

## References

[1] Lennon V. A. Wingerchuk D. M. Kryzer T. J. Pittock S. J. Lucchinetti C. F. Fujihara K. Nakashima I. Weinshenker B. G. (2004) A serum autoantibody marker of neuromyelitis optica: distinction from multiple sclerosis. Lancet 364, 2106–2112 15589308 10.1016/S0140-6736(04)17551-X

[2] Lennon V. A. Kryzer T. J. Pittock S. J. Verkman A. S. Hinson S. R. (2005) IgG marker of optic-spinal multiple sclerosis binds to the aquaporin-4 water channel. J. Exp. Med. 202, 473–477 16087714 10.1084/jem.20050304 PMC2212860

[3] Bennett J. L. Lam C. Kalluri S. R. Saikali P. Bautista K. Dupree C. Glogowska M. Case D. Antel J. P. Owens G. P. Gilden D. Nessler S. Stadelmann C. Hemmer B. (2009) Intrathecal pathogenic anti-aquaporin-4 antibodies in early neuromyelitis optica. Ann. Neurol. 66, 617–629 19938104 10.1002/ana.21802 PMC3180961

[4] Tradtrantip L. Zhang H. Saadoun S. Phuan P. W. Lam C. Papadopoulos M. C. Bennett J. L. Verkman A. S. (2012) Anti-aquaporin-4 monoclonal antibody blocker therapy for neuromyelitis optica. Ann. Neurol. 71, 314–322 22271321 10.1002/ana.22657 PMC3314396

[5] Zhang H. Bennett J. L. Verkman A. S. (2011) *Ex vivo* spinal cord slice model of neuromyelitis optica reveals novel immunopathogenic mechanisms. Ann. Neurol. 70, 943–954 22069219 10.1002/ana.22551 PMC3319401

[6] Bradl M. Misu T. Takahashi T. Watanabe M. Mader S. Reindl M. Adzemovic M. Bauer J. Berger T. Fujihara K. Itoyama Y. Lassmann H. (2009) Neuromyelitis optica: pathogenicity of patient immunoglobulin *in vivo*. Ann. Neurol. 66, 630–643 19937948 10.1002/ana.21837

[7] Saadoun S. Waters P. Bell B. A. Vincent A. Verkman A. S. Papadopoulos M. C. (2010) Intra-cerebral injection of neuromyelitis optica immunoglobulin G and human complement produces neuromyelitis optica lesions in mice. Brain 133, 349–361 20047900 10.1093/brain/awp309 PMC2822632

[8] Verbavatz J. M. Ma T. Gobin R. Verkman A. S. (1997) Absence of orthogonal arrays in kidney, brain and muscle from transgenic knockout mice lacking water channel aquaporin-4. J. Cell Sci. 110, 2855–2860 9427293 10.1242/jcs.110.22.2855

[9] Yang B. Brown D. Verkman A. S. (1996) The mercurial insensitive water channel (AQP-4) forms orthogonal arrays in stably transfected Chinese hamster ovary cells. J. Biol. Chem. 271, 4577–4580 8617713

[10] Crane J. M. Lam C. Rossi A. Gupta T. Bennett J. L. Verkman A. S. (2011) Binding affinity and specificity of neuromyelitis optica autoantibodies to aquaporin-4 M1/M23 isoforms and orthogonal arrays. J. Biol. Chem. 286, 16516–16524 21454592 10.1074/jbc.M111.227298 PMC3091256

[11] Phuan P. W. Ratelade J. Rossi A. Tradtrantip L. Verkman A. S. (2012) Complement-dependent cytotoxicity in neuromyelitis optica requires aquaporin-4 protein assembly in orthogonal arrays. J. Biol. Chem. 287, 13829–13839 22393049 10.1074/jbc.M112.344325 PMC3340190

[12] Pisani F. Mastrototaro M. Rossi A. Nicchia G. P. Tortorella C. Ruggieri M. Trojano M. Frigeri A. Svelto M. (2011) Identification of two major conformational aquaporin-4 epitopes for neuromyelitis optica autoantibody binding. J. Biol. Chem. 286, 9216–9224 21212277 10.1074/jbc.M110.123000 PMC3059066

[13] Iorio R. Fryer J. P. Hinson S. R. Fallier-Becker P. Wolburg H. Pittock S. J. Lennon V. A. (2013) Astrocytic autoantibody of neuromyelitis optica (NMO-IgG) binds to aquaporin-4 extracellular loops, monomers, tetramers, and high order arrays. J. Autoimmun. 40, 21–27 22906356 10.1016/j.jaut.2012.07.008 PMC3509259

[14] Pisani F. Mola M. G. Simone L. Rosito S. Alberga D. Mangiatordi G. F. Lattanzi G. Nicolotti O. Frigeri A. Svelto M. Nicchia G. P. (2014) Identification of a point mutation impairing the binding between aquaporin-4 and neuromyelitis optica autoantibodies. J. Biol. Chem. 289, 30578–30589 25239624 10.1074/jbc.M114.582221 PMC4215237

[15] Owens G. P. Ritchie A. M. Burgoon M. P. Williamson R. A. Corboy J. R. Gilden D. H. (2003) Single-cell repertoire analysis demonstrates that clonal expansion is a prominent feature of the B cell response in multiple sclerosis cerebrospinal fluid. J. Immunol. 171, 2725–2733 12928426 10.4049/jimmunol.171.5.2725

[16] Ho J. D. Yeh R. Sandstrom A. Chorny I. Harries W. E. Robbins R. A. Miercke L. J. Stroud R. M. (2009) Crystal structure of human aquaporin 4 at 1.8 A and its mechanism of conductance. Proc. Natl. Acad. Sci. U.S.A. 106, 7437–7442 19383790 10.1073/pnas.0902725106 PMC2678640

[17] Lucchinetti C. F. Bruck W. Lassmann H. (2004) Evidence for pathogenic heterogeneity in multiple sclerosis. Ann. Neurol. 56, 308 15293289 10.1002/ana.20182

[18] Parratt J. D. Prineas J. W. (2010) Neuromyelitis optica: a demyelinating disease characterized by acute destruction and regeneration of perivascular astrocytes. Mult. Scler. 16, 1156–1172 20823059 10.1177/1352458510382324

[19] Kowarik M. C. Dzieciatkowska M. Wemlinger S. Ritchie A. M. Hemmer B. Owens G. P. Bennett J. L. (2015) The cerebrospinal fluid immunoglobulin transcriptome and proteome in neuromyelitis optica reveals central nervous system-specific B cell populations. J. Neuroinflammation 12, 19 25626447 10.1186/s12974-015-0240-9 PMC4323273

[20] Wrzos C. Winkler A. Metz I. Kayser D. M. Thal D. R. Wegner C. Brück W. Nessler S. Bennett J. L. Stadelmann C. (2014) Early loss of oligodendrocytes in human and experimental neuromyelitis optica lesions. Acta Neuropathologica 127, 523–538 24292009 10.1007/s00401-013-1220-8 PMC4229038

[21] Kampylafka E. I. Routsias J. G. Alexopoulos H. Dalakas M. C. Moutsopoulos H. M. Tzioufas A. G. (2011) Fine specificity of antibodies against AQP4: epitope mapping reveals intracellular epitopes. J. Autoimmun. 36, 221–227 21333492 10.1016/j.jaut.2011.01.004

[22] Nicchia G. P. Mastrototaro M. Rossi A. Pisani F. Tortorella C. Ruggieri M. Lia A. Trojano M. Frigeri A. Svelto M. (2009) Aquaporin-4 orthogonal arrays of particles are the target for neuromyelitis optica autoantibodies. Glia 57, 1363–1373 19229993 10.1002/glia.20855

